# Immune Checkpoint Inhibitor-Induced Primary Hyperparathyroidism in a Small-Cell Lung Cancer Patient: A Case Report

**DOI:** 10.3390/medicina59020215

**Published:** 2023-01-22

**Authors:** Ying Zhang, Yangang Cui, Yan Li, Lei Cong

**Affiliations:** 1 Department of Oncology, Shandong Provincial Hospital Affiliated to Shandong First Medical University, Jinan 250021, China; 2 Department of Oncology, Shandong Provincial Hospital, Cheeloo College of Medicine, Shandong University, Jinan 250021, China

**Keywords:** immune checkpoint inhibitors, immune-related adverse events, hyperparathyroidism, hypercalcemia, hypophosphatemia

## Abstract

Immunotherapy is increasingly being used in the treatment of tumors. Adverse effects, however, are not uncommon, with the most common immune-related adverse events (IrAEs) including rash, fatigue, anemia, diarrhea, constipation, and elevated transaminase, among others. Rare IrAEs, which may include thrombocytopenia, hypoparathyroidism, pancreatitis glomerulonephritis, Guillain-Barré syndrome, and celiac disease, may also present. Immune checkpoint inhibitor (ICI)-induced primary hyperparathyroidism (PHPT) has not yet been reported on, and no research currently exists regarding its pathogenesis. We describe how a 50-year-old man diagnosed with advanced small-cell lung cancer (SCLC) developed severe PHPT after receiving the programmed cell death (PD)-1 inhibitor camrelizumab. The patient eventually died of respiratory failure and a progressive malignancy. We speculate that the hypercalcemia and hypophosphatemia observed in this case were secondary to ICI-induced PHPT. Although fatal PHPT is rare, early intervention may reduce the risk of future complications. Therefore, further exploration of the underlying mechanisms is needed to guide solutions.

## 1. Introduction

The immune system and tumor cells are continuously, dynamically processed. Owing to immune escape, tumor cells can proliferate uncontrollably [1]. Traditional radiotherapy and chemotherapy not only kill tumors but also cause irreversible damage to normal organs and tissues. In patients with various histological metastatic cancers, immunotherapy functions by stimulating, augmenting, inhibiting, or desensitizing the immune system [2]. Harm to normal tissues and organs is largely avoided by focusing treatment on the immune system and mobilizing immune cells to recognize and remove tumor cells. Immunotherapy also has the potential to improve the quality of life of patients. For example, immune checkpoint inhibitors (ICIs), such as anti-programmed cell death 1 antibodies, anti-programmed cell death 1 ligand 1 antibodies, and anti-cytotoxic T lymphocyte antigen 4 antibodies, work by blocking programmed cell death 1 (PD-1), programmed cell death ligand 1 (PD-L1), and cytotoxic T lymphocyte antigen 4 (CTLA-4) to overcome or reduce tumor-induced immunosuppression, therefore allowing for immune-mediated tumor clearance [3].

IrAEs differ from the traditional toxicities associated with chemotherapy and radiotherapy. They are regarded as non-targeted effects of immune system overactivation [4]. Patients who respond well to ICIs are more likely to develop autoimmune toxicity [5]. The rate of fatal adverse events associated with ICIs is estimated to be between 0.3 and 1.3% [6]. The incidence of IrAEs is highest in patients who receive CTLA-4 ICIs, and lowest in patients who receive PD-L1 ICIs [7]. PD-1 ICIs are more likely to cause adverse reactions ≥ grade 3 compared with PD-L1 ICIs [8]. Moreover, patients on PD-1 ICIs have a higher risk of hypothyroidism, pneumonitis, colitis, and hypophysitis [9]. Combination therapy has been shown to have more negative consequences compared with monotherapy [8,10,11,12,13,14,15,16].

Endocrine diseases caused by ICIs are also common. Thyroiditis, hypophysitis, hypoparathyroidism, and diabetes are endocrine-related adverse events, all of which have been reported [17,18,19,20]. Owing to the non-specific and diverse nature of symptoms associated with ICI-induced endocrinopathies, timely diagnosis requires a high index of suspicion. PHPT caused by ICIs is rare and has not yet been reported. We present a case of PTPH caused by the PD-1 inhibitor camrelizumab. We also review the epidemiology, treatment, and prognosis of ICI-induced endocrine disorders caused by ICIs.

## 2. Case Report

The patient was a 50-year-old man with a 5-year history of hypertension, 30-year history of smoking, and a 20-year history of drinking. He had no history of immune diseases, and physical examination showed no specific positive symptoms of this.

The patient was hospitalized and diagnosed with advanced SCLC on May 10, 2019, with metastases to the hilum, mediastinum, and left adrenal gland. Immunohistochemical examination revealed synaptophysin (Syn) (+), chromogranin A (CgA) (+), and Ki67 (+, 30%). Tumor marker examination showed that Neuron Specific Enolase (NSE) was 30.42 (0–16.3) ng/mL and gastrin-releasing peptide (pro-GRP) was 298.10 (0–50) pg/mL. His initial biochemical test results were within normal limits, including full blood count and renal and liver profiles. Cranial magnetic resonance imaging (MRI) ruled out brain metastases and other brain diseases. After four cycles of etoposide and nedaplatin therapy, the patient’s lesions of the hilum, the enlarged lymph nodes of the mediastinum and the bilateral hilum were smaller than before. Compared to the baseline lesion, the total size of the target lesion was less than 30%. We evaluated that the patient’s condition remained stable according to the revised RECIST guideline (version 1.1) [21]. The patient was then treated with radiotherapy: 60 Gy/2Gy*30F for lung lesions. Unfortunately, the patient developed brain metastases after lung lesions radiotherapy. He was treated with brain radiotherapy: 30 Gy/3Gy*10F.

The patient developed brain metastases soon after chemotherapy and radiation. Considering his rapidly progressing disease and the poor efficacy of second-line chemotherapy, chemotherapy was no longer recommended. At that time, PD-L1 was not available in China, and PD-1 was actively selected for financial reasons. The PASSION study on camrelizumab for extensive-stage SCLC demonstrated that patients with sensitive and drug-resistant relapses could benefit from its use [22]. We used camrelizumab (200 mg, every three weeks) in combination with anlotinib (8 mg daily for 2 weeks, followed by a 1-week break) beginning from 10 January 2020, owing to the poor effect of PD-1 alone. Laboratory tests for the patient’s immunological indices were normal, and no symptoms of autoimmunity were observed prior to the commencement of this treatment. The patient’s condition was still stable during this period, and his overall health and quality of life improved significantly.

On 24 September 2020, the patient exhibited symptoms of dysphagia, trance, impaired limb mobility, and grade 3 muscle strength. He displayed a poor mental state, inappetence, glossolalia, urination difficulties, and severe dependence on self-care 10 days prior to this. Laboratory tests (25 September 2020) showed that his levels of adrenocorticotropic hormone (ACTH), cortisol (COR), and ferritin (FER) were 109.7 (normal ranges: 7.2–63.3) pg/mL, 999.4 (normal ranges: 133–537) nmol/L, and 1111.6 (normal ranges: 133–537) ng/mL, respectively. Renal and liver profiles showed no obvious abnormalities. The abnormal elevation of COR was thought to be due to ACTH-dependent Cushing syndrome caused by Camrelizumab. This adverse event has been reported previously [23]. Hence, we highly suspect that the patient had endocrine IrAEs. Since the patient displayed a poor mental state, a cranial MRI could not be performed. Immunological damage caused by camrelizumab and brain metastasis were not excluded. He was treated with 80 mg of methylprednisolone once daily after an assessment of immunological damage. In addition, the patient was treated with 40 mg of pantoprazole sodium for injection once daily, 250 mL of medium and long-chain fat emulsion injection once daily, and 250 mL of compound amino acid injection once daily.

**Figure 1:**
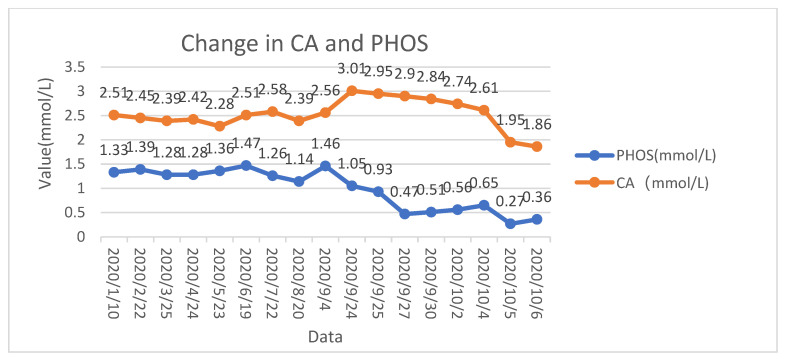
Serum phosphorus and calcium levels of patients during treatment with Camrelizumab.

The patient’s calcium levels and phosphorus levels had been normal on previous admissions. It was noteworthy that the patient’s calcium levels at this admission was 3.01 (normal ranges: 2.2–2.7) mmol/L, with albumin-adjusted calcium of 3.02 mmol/L. After three days (27 September 2020) of methylprednisolone therapy, his calcium was 2.9 mmol/L; albumin-adjusted calcium was 2.96 mmol/L; phosphorus was 0.47 mmol/L. We then tested his parathyroid hormone (PTH) level, and it was 300.5 (normal ranges: 15–65) pg/mL. The 25-hydroxyvitamin D level was 65 ng/mL (normal ranges: 30–100) ng/mL. We considered hypercalcemia to be caused by elevated PTH for the following reasons: (I) he had no history of calcium disorders, Sarcoidosis-like granuloma, kidney stones, fractures, osteoporosis, and hypothyroidism. (II) lumbar enhanced CT (29 August 2020) and a bone scan (6 September 2020) showed no bone metastasis. (III) at the time of admission, renal and liver profiles showed no obvious abnormalities. (IV) the patient had no symptoms of vomiting or diarrhea, and full blood count and normal urine output were normal. Hence, it ruled out hypercalcemia due to immobilization and hemoconcentration. The diagnosis of PHPT should be considered when the patient has hypercalcemia and the PTH level is higher than normal, according to the National Institute for Health and Care Excellence (NICE) guidelines [24]. At the time, camrelizumab was thought to have caused PHPT, which then further led to hypercalcemia and hypophosphatemia. The patient continued to be treated with methylprednisolone (80 mg once daily) and composite potassium hydrogen phosphate injection (2 mL once daily). His PTH subsequently dropped to 217 pg/mL, calcium levels dropped to 2.84 mmol/L, albumin-adjusted calcium levels dropped to 2.90 mmol/L, and phosphorus rose to 0.51 mmol/L (30 September 2020). The indicators of this patient improved. After two days (2 October 2020), his PTH level was 123 pg/mL. On 4 October 2020, his PTH levels subsequently dropped to 70 pg/mL, which is close to normal. At this time, his levels of calcium, albumin-adjusted calcium, and phosphorus were 2.61 mmol/L, 2.69 mmol/L, and 0.65 mmol/L. As the patient was in the terminal stage, the patient’s calcium and phosphorus began to decrease due to cachexia. The results from the analysis of the effective methylprednisolone treatment provided evidence for the diagnosis of PHPT due to an ICI. The levels of calcium and phosphorus in the patient’s body are shown in Figure 1.

As he was completely bedridden for a significant period of time and his muscles were paralyzed, the patient developed a secondary pulmonary infection. The patient went into a coma on 6 October 2020. Unfortunately, he died of respiratory failure on 8 October 2020.

The patient had received ICI for approximately 8 months, with a 17-month survival time. Patients with advanced SCLC have an average 10-month and 13-month survival time with and without administration of PD-1, respectively. The patient’s overall survival (OS) was significantly longer than the median OS for patients with SCLC. Secondly, the patient’s quality of life was considerably improved after PD-1 treatment compared to conventional chemotherapy, and his progression-free survival (PFS) was significantly longer than that of patients who undergo only first-line chemotherapy. This indicated that PD-1 was effective. Clinical examination showed that the patient was delirious and had a limb mobility disorder. Zhang et al. [25] showed that FER could be used as a diagnostic and differential diagnostic marker for IrAEs in patients with ICI treatment. The patient’s ferritin was abnormally elevated. Hence, he likely had immunological damage. During the course of treatment with methylprednisolone, the patient’s ACTH and COR also dropped significantly, so we became more suspicious of an endocrine disorder caused by ICI. PTH and albumin-adjusted calcium levels were greatly increased, while phosphorus levels were significantly decreased. Thus, we postulate that the diagnosis of PHPT was secondary to camrelizumab administration. These indicators improved after treatment with methylprednisolone. Our final diagnosis was that of camrelizumab-induced PHPT. In the advanced stages of his illness, the patient suffered from a pulmonary infection and subsequently died of respiratory failure.

## 3. Discussion

PHPT is more common in those with benign parathyroid tissue overgrowth [26]. Parathyroid adenomas are the most common cause of PHPT [27]. PHPT caused by ICIs is rare and has not yet been reported. To our knowledge, this is the first reported case of PHPT caused by camrelizumab therapy. Although our patient did not undergo parathyroid imaging, abnormal laboratory tests and effective methylprednisolone therapy provided the basis for diagnosis. PHPT occurs due to an increase in the number and/or volume of parathyroid cells and/or a decrease in calcium-sensing receptor (CaSR) expression [28]. However, we did not find any relevant literature on the pathophysiological mechanisms of ICI-induced PHPT. Hypercalcemia and abnormal PTH levels are the primary features of PHPT. PHPT can cause skeletal, renal, neurological, and cardiovascular diseases [29]. This patient not only exhibited neuromuscular weakness and impaired limb mobility but also presented with dysphagia and trance. However, these symptoms are not clinical manifestations specific to PHPT. PTH levels in PHPT are usually within twice the upper normal limit [30]. However, PTH levels in this patient were close to five times the upper normal limit. This suggests that ICI-induced PHPT may be serious. Parathyroidectomy (PTX) is recommended in patients with symptomatic hyperparathyroidism. Hydration and antiresorptive drugs (such as calcitonin or intravenous bisphosphonates) are also recommended treatments [26,31]. As the patient’s PHPT was caused by ICIs and his general condition was poor, we treated the patient with steroids in reference to the management of IrAEs. In addition, hypercalcemia can be caused by endocrine disease-related conditions, sarcoid-like granuloma, humoral hypercalcemia due to parathyroid-related hormones, and hyperprogressive diseases [32]. When serum calcium levels are elevated, a thorough examination of the patient, including PTH measurement, is necessary. The case we report may provide important lessons for oncologists in the diagnosis and treatment of ICI-induced PHPT.

The frequency of endocrine toxicity was considerably lower in the oldest cohort compared with the youngest cohort, according to a retrospective multicenter investigation of 448 patients treated with ICIs. We found no substantial increase in adverse events in older individuals, and all levels of toxicity were significantly associated with treatment type [33]. In addition, ICI-related endocrinopathies were reported slightly less frequently in males than in females, though this difference was not statistically significant [14]. The incidence of endocrine irAEs varies according to the type of ICI used [11]. A combination of antiCTLA-4 and anti-PD-1 therapy is associated with the highest incidence of ICI-related endocrinopathies [15]. The clinical symptoms of endocrine irAEs are non-specific and may include fever, gastrointestinal abnormalities, disturbance of consciousness, coma, and electrolyte imbalance, among others [34]. At present, steroids are the most frequently used drugs for the endocrine treatment of IrAEs. For severe and steroid-resistant IrAEs, biopsy samples should be obtained, and immune cell infiltration should be explored to suppress critical inflammatory components implicated in the pathophysiology of IrAEs [4,35]. According to the National Cancer Institute’s Common Terminology Criteria for Adverse Events (CTCAE) classification, steroid therapy can be used for grade 1 and 2 IrAEs, and antitumor therapy with ICIs can be continued after symptom improvement. Nonetheless, grade 3 or 4 irAEs can be severe or fatal. Thus, the use of this may need to be stopped [2]. Additionally, the endocrine system is the most commonly IrAE-induced irreversibly damaged organ system, and immune-related endocrine toxicity is largely irreversible in 50% of patients [36]. Since it can lead to the permanent destruction of the endocrine organs and rule out their ability to resume steroid production, long-term treatment is frequently needed [11,37]. According to the majority of studies, patients who experienced IrAEs displayed significantly better PFS, OS, and total response rates than those who did not [5,38]. Steroid treatment administered at the beginning of immunotherapy may adversely affect PFS and OS [39].

## 4. Conclusions

In conclusion, we present a complex case of ICI-induced PHPT in a patient with metastatic SCLC. ICI-induced PHPT is uncommon in clinical settings. However, immune-related PHPT should not be neglected during treatment with ICIs. More clinical evidence is needed to determine the frequency, timing, severity, and efficacy of hyperparathyroidism treatment. Patient mortality can be greatly reduced if these can be identified in time, allowing patients to receive maximum benefit from immunotherapy. Early diagnosis of symptoms, identification of indicators, laboratory testing, and imaging examinations are critical for reducing the risk of silently affected organs and/or delayed incidence. With the continued development of medicine, specific regulation of ICIs against malignancies has the potential to improve patient outcomes.

## Data Availability

Not applicable.
